# Exertional Dyspnea Incidentally Diagnosed as Sarcoidosis: A Teaching Hospital Experience

**DOI:** 10.1155/2023/8689352

**Published:** 2023-09-05

**Authors:** Melvina Nartey, Kofi Ulzen-Appiah

**Affiliations:** ^1^Department of Pathology, School of Medical Sciences, University of Cape Coast, Cape Coast Teaching Hospital, Cape Coast, Ghana; ^2^ACT Pathology Consult, Cape Coast, Ghana

## Abstract

**Background:**

Sarcoidosis is a complex disease with nonspecific etiology and clinical presentation. Its diagnosis is often delayed due to the absence of a single specific investigation modality. A multidisciplinary approach is necessary for its diagnosis. *Report*. A 49-year-old male presented with recurrent dyspnea on exertion, easy fatigue, and chest pain after several visits to different health facilities over 5 months. A diagnosis of pulmonary sarcoidosis was made after a series of laboratory and imaging investigations were done revealing bilateral reticonodular opacifications, noncaseating granulomata, elevated serum ACE and calcium levels consistent with sarcoidosis.

**Conclusion:**

Sarcoidosis, although a rare presentation in our setting, may easily be overlooked or misdiagnosed if a holistic or multidisciplinary approach is not employed in its diagnosis. Nonspecificity of symptoms contributes to the delayed diagnosis.

## 1. Introduction

Sarcoidosis is a granulomatous disease that commonly affects the lungs, and its worldwide prevalence is estimated to be between 4 and 6 in 100,000 [1]. Some believe that sarcoidosis is rare in Africa [2] on account of the relatively low rate of reporting of such cases [3].

Data on the disease burden of sarcoidosis in Ghana is not readily available. However, a number of cases have been reported such as that by Bajantri et al. who reported on a 53-year-old woman with neurosarcoidosis [4] and two cases of cardiac sarcoidosis in a 51-year-old man and a 58-year-old woman reported by Afriyie-Mensah et al. [5]. It is more common in females [1], and the age at diagnosis is usually between 40 and 55 years [1]. Sarcoidosis is uncommon in people less than 15 years of age and greater than 70 years of age [6].

The elusiveness of the etiology makes the condition a subject of ongoing research [1]. Several theories abound about the cause of sarcoidosis. One such hypothesis is exposure to several different antigens, a genetic predisposition, and the appropriate immunological environment contribute to developing the disease [7].

Pulmonary sarcoidosis is believed to be caused by immune dysregulation in individuals with certain HLA genotypes such as HLA DR/DG(MHC2) [8]. A large number of CD4+ T cells accumulate in the alveoli, which leads to the production of T helper-1 cytokines such as IL-2 and TNF-*α* leading to massive macrophage activation. Other cells involved include monocytes and lymphocytes. Cytokines and chemokines also play a role in the pathogenesis of pulmonary sarcoidosis [8].

Sarcoidosis presents with several clinical manifestations and may affect multiple organs such as the liver, spleen, kidney, heart, brain, and even the skin [9].

Although no clinical feature is pathognomonic for sarcoidosis, patients may present with complaints of a gradual onset of respiratory symptoms and constitutional symptoms which include dyspnea on exertion, a common symptom reported in about 55% of cases, fatigue, wheezing, arthralgia, and uveitis which is an infrequent symptom reported in 16% of cases [10]. Sarcoidosis may affect the pleura leading to pleural effusion, hemothorax, chylothorax, and pulmonary nodules, and may affect the airways causing narrowing of the bronchi with atelectasis [11].

Neurosarcoidosis presents symptoms such as meningitis, seizures, neuroendocrine symptoms, and neuropsychiatric symptoms (4).

Cardiac sarcoidosis presents with cardiomyopathy associated with progressive heart failure symptoms such as chest pain, cough, peripheral edema, palpitations, and syncope [12]. In the skin, it might present as a scar sarcoid in a long-standing tribal mark [13].

Hepatic sarcoidosis may present as cirrhosis, portal hypertension, and cholestasis [14].

Sarcoidosis presents in the kidney with symptoms such as nephrolithiasis on account of the hypercalciuric state of the blood [15].

Confirmation of the diagnosis is on biopsy, which will show noncaseating granulomas with an absence of central necrosis. Asteroid bodies within giant cells may be seen in about 60% of the cases [16].

Serum angiotensin-converting enzyme (ACE) may be elevated in up to 75% of cases; however, it is not specific for sarcoidosis [17]. Serum calcium greater than 3 mmol/l is seen in 5-10% of sarcoidosis cases [18].

Imaging techniques may include a plain erect chest X-ray for lung involvement which is useful in staging; a chest CT scan is helpful for detecting specific complications of sarcoidosis such as aspergilloma, bronchiectasis, and pulmonary fibrosis [17].

A PET scan and MRI are effective in detecting the disease in all other organs involved and help with finding good areas to biopsy [17].

A gallium scan is helpful in parotid and lacrimal involvement, and a thallium scan is helpful in differentiating cardiac sarcoidosis from coronary artery disease [17].

Long-term management with steroids is the first line of treatment [19, 20]. However, because of the complications of long-term steroid use and in people with steroid resistance, certain other drugs may be used [20]. Complications of steroid treatment include steroid-induced immune suppression leading to opportunistic infections such as nocardiosis, pneumocystis, and histoplasmosis [21]. Sarcoidosis is also strongly associated with depression, especially in female patients [4].

Differential diagnosis of sarcoidosis includes cat-scratch disease, berylliosis, toxoplasmosis, coccidioidomycosis, tuberculosis, and malignancy [22].

We highlight a case of a 49-year-old man who presented to our outpatient department with complaints of dyspnea on exertion and was diagnosed with stage 3 pulmonary sarcoidosis following laboratory, imaging, and histopathological investigations.

## 2. Case Presentation

**Figure 1:**
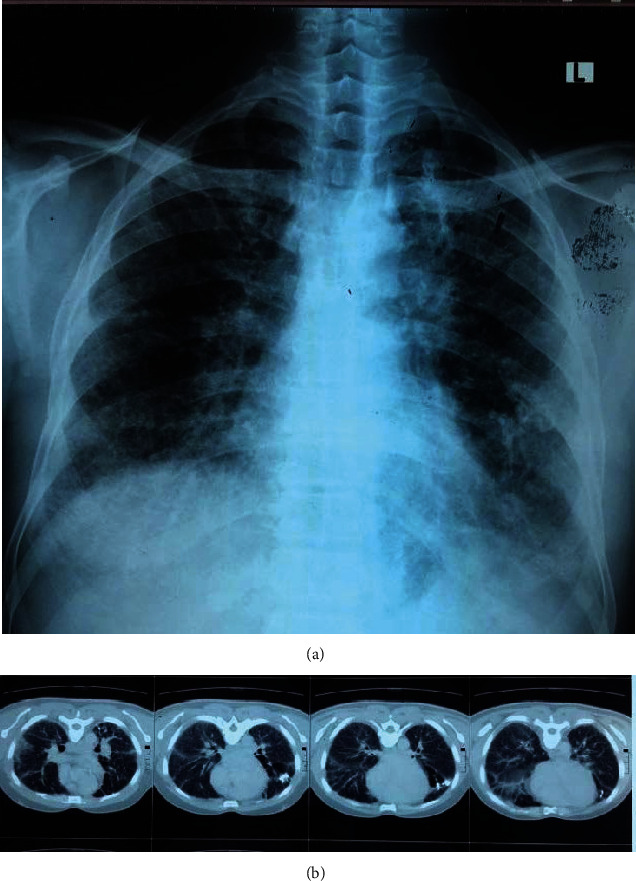
(a) Chest X-ray showing diffuse bilateral reticulonodular changes. (b) Chest CT scan showing diffuse bilateral reticulonodular opacities with multiple left lung calcifications and cardiomegaly with dilated pulmonary trunk.

A 49-year-old male bus driver presented with a five-month history of dyspnea on exertion of insidious onset associated with easy fatiguability and non-radiating central chest pain with associated palpitations. The dyspnea waxed and waned but never completely resolved. He had no history of chronic cough. On examination, he was stable, with blood pressure of 132/87mmHg, pulse 99b/min, temperature 36.2°C, and blood oxygen saturation on room air of 96%. On chest examination, air entry was reduced bilaterally, but breath sounds were normal. The cardiovascular system exam disclosed an apex beat located in the left 5th intercostal space, anterior axillary line; heart sounds were normal with no murmurs. A diagnosis of respiratory tract infection was made. The initial plan was to do a full blood count, urine routine exam, renal and liver function tests, lipid profile, chest X-ray, and an electrocardiogram (ECG). He presented the next day with the lab (Table 1), ECG, and chest X-ray results. Chest X-ray showed diffuse bilateral reticulonodular infiltrates without bilateral hilar lymphadenopathy (Figure 1(a)) with differentials of pulmonary tuberculosis with fibrosis, sarcoidosis, and pneumoconiosis.

The electrocardiogram showed sinus rhythm and no abnormalities. Subsequently, the plan was to request a gene expert for acid-fast bacilli. He presented a day after with a negative gene expert for Mycobacterium tuberculosis.

A computerized tomography (CT) scan of the chest disclosed, diffuse bilateral reticulonodular, predominantly reticular opacities with multiple left lung calcifications. In a focus, the nodules coalesced to form a macronodule with central calcification. There was no associated traction bronchiectasis, and the hilar was normal. The trachea was central with normal caliber. Other significant findings included cardiomegaly and a dilated pulmonary trunk (Figure 1(b)). Suggested differential diagnoses included idiopathic pulmonary fibrosis with pulmonary hypertension, lymphangitis, carcinomatosis, and stage 3 sarcoidosis. A CT-guided tru-cut biopsy of the lung lesion was done.

**Figure 2:**
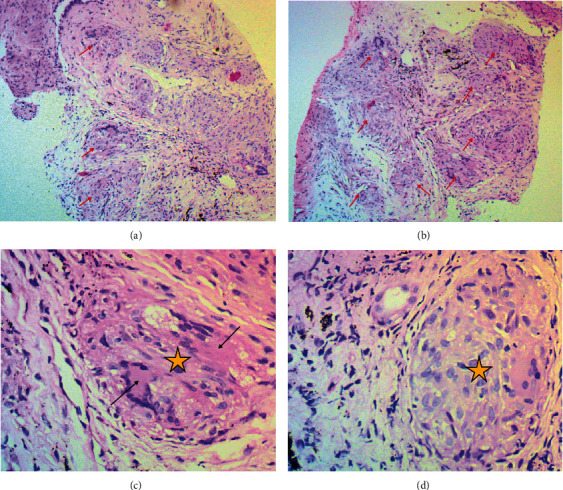
(a, b) *H&E ×100* shows effacement of lung tissue by multiple variably sized naked noncaseating granulomata (red arrows) with surrounding fibrosis containing lymphocytic cells. (c–d) *H&E ×400* shows a high-power view of noncaseating granulomata (orange star) with multinucleate Langhans giant cells (black arrow).

At the histopathology lab, two tru-cut tissue cores were examined, measuring 20 mm on average. Hematoxylin and eosin-stained slides showed effacement of lung architecture by multiple naked noncaseating granulomata, a few associated with Langhans' giant cells (Figures 2(a)–2(d)) The features were suggestive of a noncaseating granulomatous inflammation with differentials of sarcoidosis, foreign body inhalation, and parasitic infection.

Serum calcium and angiotensin-converting enzyme (ACE) levels were suggested, and if high, to favor a diagnosis of sarcoidosis. A clinical-radiologic correlation was recommended.

Further history taken revealed no prior history of penile rashes or ulcers, no history of terminal hematuria, and no recent close contact with a cat. Lung spirometry revealed severe restriction with no significant bronchodilator response. The test result showed elevated serum calcium and ACE level (Table 1).

A diagnosis of stage 3 pulmonary sarcoidosis was made due to the presence of granulomata microscopically, the presence of pulmonary reticulonodular infiltrates without bilateral hilar lymphadenopathy, and scant to absent fibrosis on chest imaging. He was commenced on prednisolone at 60 mg daily to be continued in tapering doses for a period of 1 year. He reported 2 months after starting medication and is doing well.

## 3. Discussion

Sarcoidosis is a multisystem disease that is common in people of African descent, and seen in individuals who work in the agricultural sector and exposed to insecticides or in people who work in an environment where they may be exposed to microbial bioaerosols such as mold or mildew [2] and to metal such as beryllium or to smoke [3]. Sarcoidosis is more common in women [1, 23] than in men; however, women are more likely to be asymptomatic in the expression of the disease. Our client is a man of Ghanaian descent who works as a bus driver which is not a recognized profession associated with sarcoidosis, and not involved in farming activities. However, his profession as a driver may have exposed him to mold, mildew, metal dust, and smoke. His age also falls within the bracket of the average age of onset.

A delay in diagnosis is more common in pulmonary sarcoidosis than in other sites such as cutaneous sarcoidosis [24],because the respiratory symptoms are usually mistaken for other conditions, most notably tuberculosis [25]. Our client reported with a 5-month history of worsening symptoms after being managed at several other facilities.

Sarcoidosis commonly presents with constitutional symptoms such as joint pain, loss of appetite, fever, fatigue, general body weakness, myalgia, and cognitive failure [26]. However, our client did not complain of these symptoms.

A constellation of symptoms called Lofgren's syndrome made up of erythema nodosum, migratory polyarthritis, and fever, which occurs in younger patients, is distinct for sarcoidosis and is associated with a favorable prognosis [27]. Patients with sarcoidosis may also present with Heerfordt's syndrome, which includes symptoms such as facial palsy, parotid enlargement, and uveitis [28]. Our client did not exhibit either of these syndromes.

There is no particular test that confirms sarcoidosis; nevertheless, a combination of radiographic imaging findings, clinical symptoms, laboratory findings and histological picture all contribute to making the diagnosis [29]. Increased ACE activity is also helpful in diagnosis [30]. Serum calcium levels above 3 mmol/L and urine calcium above 0.10 mmol/L/kg per day may be indicative of sarcoidosis [31]. Typical laboratory investigations such as ACE, lysozyme, and calcium are not always specific [31] and may be elevated in sarcoidosis as seen in our case who had elevated serum calcium and ACE above normal levels. Elevated serum ACE may be seen in coccidioidomycosis, diabetes mellitus, leprosy, lung cancer, hyperthyroidism, and Gaucher's disease [32].

Histopathological diagnosis of sarcoidosis is defined by noncaseating granulomas, occasional ring fibrosis, and fibrinoid necrosis. Other inclusion bodies such as Schaumann bodies, Langhans giant cells, birefringent concretions, asteroid bodies, and Hamazaki-Wesenberg's bodies may be seen [33]. Noncaseating granulomas are also seen in histoplasmosis, coccidioidomycosis, tuberculosis, schistosomiasis, leprosy, and syphilis [16]. Our case showed noncaseating naked granulomata associated with occasional Langhans giant cells.

Treatment includes a multidisciplinary approach, especially in systemic disease made up of a specialized interdisciplinary team led by a primary care physician [26].

Medical treatment of pulmonary sarcoidosis is only indicated in people who have markedly reduced pulmonary function with a significant reduction in physical reserve [19]. Prednisone is started at an initial dose of 0.5 mg per kg body weight to be tapered during the treatment. The period of treatment with steroids is recommended to be at least 6 months but may be extended for a year or more. Our client was commenced on prednisolone at 60 mg daily to be tapered during a one-year period, due to the presence of multiple noncaseating granulomas with scant to absent fibrosis which would respond positively to administered steroids.

Adverse effects of long-term steroid use include susceptibility to infection, osteoporosis, fractures, hyperglycemia, and obesity [36]. In cases of steroid resistance, a weekly low-dose methotrexate therapy, azathioprine, infliximab, and leflunomide may be used [19, 20].

The recommended period for follow-up is 6-12 months. Pulmonary function tests, especially vital capacity and chest X-ray, can be used to assess for resolution of symptoms or for progression of symptoms [26]. It is also important to assess for the involvement of other systems which were not previously involved at the onset of treatment.

The classification of sarcoidosis will depend on the outcome of the treatment. It is termed acute if the symptoms resolve between 2 and 5 years after the start of treatment. If symptoms persist after 5 years of treatment, it is termed chronic, and the classification of refractory sarcoidosis is used if symptoms worsen after the start of treatment [19].

## 4. Conclusion

Delayed diagnosis is likely and seen in about 50% of cases on account of nonspecificity of the clinical signs and symptoms; however, a high index of suspicion should be entertained and a holistic multidisciplinary approach employed for its diagnosis in a low-resource setting.

## Figures and Tables

**Table 1:** A summary of laboratory investigations done.

Investigations	Indices	Value	Reference ranges
Full blood count	Hemoglobin count	13.8 g/dL	13.2-16.6 g/dL
	White cell count	7.3 × 10^3^/*μ*L	4.5 − 11.0 × 10^3^/*μ*L
	Platelets	501 × 10^3^/*μ*L	150 − 400 × 10^3^/*μ*L
	Eosinophils	0.37 × 10^3^/*μ*L	0 − 0.5 × 10^3^/*μ*L

Lipid profile	Cholesterol	5.64 mmol/L	<5.17 mmol/L
	Non-HDL	5.0 mmol/L	<3.37 mmol/L
	Coronary risk	8.8	
	ASCVD 10-year risk	9.5%	<7%

Urine routine exam	Leucocytes	1+	Negative
	Proteins	1+	<150 mg/dL/negative
	Calcium oxalate crystals	1+	Occasionally

Renal function test	Creatinine	185.6 *μ*mol/L	55-106 *μ*mol/L
	Urea	7.2 mmol/L	3.6-7.1 mmol/L
	eGFR	42 mmol/L	8-52 mmol/L

Serum ACE		84.7 IU/L	8-52 IU/L

Serum calcium		3.5 mmol/L	2.25-2.62 mmol/L

## References

[1] Mankad P., Mitchell B., Birnie D., Kron J. (2019). Cardiac sarcoidosis. *Current Cardiology Reports*.

[2] Tsega E., Getahun B., Teklehaimanot R. (1978). Sarcoidosis in Ethiopia. *Tubercle*.

[3] Judson M. A. (2020). Environmental risk factors for sarcoidosis. *Frontiers in Immunology*.

[4] Bajantri B., Venkatram S., Niazi M., Singh T., Diaz-Fuentes G. (2017). Case report: middle-aged woman from Ghana with unsteady gait and enlarging cerebellar mass. *Medicine*.

[5] Afriyie-Mensah J. S., Awindaogo F. R., Tagoe E. N. D., Ayetey H. (2021). Cardiac sarcoidosis: two case reports. *Clinical Case Reports*.

[6] Cozier Y. C., Arkema E. V., Rodriguez J. V., Berman J. S., Govender P. (2022). Epidemiology: solving the jigsaw puzzle. *Sarcoidosis (ERS Monograph)*.

[7] Saidha S., Sotirchos E. S., Eckstein C. (2012). Etiology of sarcoidosis: does infection play a role?. *The Yale Journal of Biology and Medicine*.

[8] Gerke A. K., Hunninghake G. (2008). The immunology of sarcoidosis. *Clinics in Chest Medicine*.

[9] Cozier Y. C. (2016). Assessing the worldwide epidemiology of sarcoidosis: challenges and future directions. *European Respiratory Journal*.

[10] Chang B., Steimel J., Moller D. R. (2001). Depression in sarcoidosis. *American Journal of Respiratory and Critical Care Medicine*.

[11] Mihailovic-Vucinic V., Jovanovic D. (2008). Pulmonary sarcoidosis. *Clinics in Chest Medicine*.

[12] Kusano K. F., Satomi K. (2016). Diagnosis and treatment of cardiac sarcoidosis. *Heart*.

[13] Alabi G. O., George A. O. (1989). Cutaneous sarcoidosis and tribal scarifications in West Africa. *International Journal of Dermatology*.

[14] Karagiannidis A., Karavalaki M., Koulaouzidis A. (2006). Hepatic sarcoidosis: Concise review. *Annals of Hepatology*.

[15] La Rochelle J. C., Coogan C. L. (2012). Urological manifestations of sarcoidosis. *The Journal of Urology*.

[16] Tana C., Donatiello I., Caputo A. (2022). Clinical features, histopathology and differential diagnosis of sarcoidosis. *Cell*.

[17] Soto-Gomez N., Peters J. I., Nambiar A. M. (2016). Diagnosis and management of sarcoidosis. *American Family Physician*.

[18] Conron M., Young C., Beynon H. L. C. (2000). Calcium metabolism in sarcoidosis and its clinical implications. *Rheumatology*.

[19] Prasse A. (2016). The diagnosis, differential diagnosis, and treatment of sarcoidosis. *Deutsches Ärzteblatt International*.

[20] Yakubu A. A., Edino S. T., Mohammed A. Z., Yakubu A. (2010). Subcutaneous sarcoidosis in a Nigerian female. *Annals of African Surgery*.

[21] Dhote R., Abad S., Valeyre D. (2009). The infectious complications of sarcoidosis. *Presse Médicale*.

[22] Baughman R. P., Costabel U., du Bois R. M. (2008). Treatment of sarcoidosis. *Clinics in Chest Medicine*.

[23] Müller-Quernheim J., Schürmann M., Hofmann S. (2008). Genetics of sarcoidosis. *Clinics in Chest Medicine*.

[24] Judson M. A., Thompson B. W., Rabin D. L. (2003). The Diagnostic Pathway to Sarcoidosis. *Chest*.

[25] Jacyk W. K. (1999). Cutaneous sarcoidosis in black South Africans. *International Journal of Dermatology*.

[26] Drent M., Costabel U., Crouser E. D., Grunewald J., Bonella F. (2021). Misconceptions regarding symptoms of sarcoidosis. *The Lancet Respiratory Medicine*.

[27] Brown F., Modi P., Tanner L. S. (2021). Lofgren Syndrome. *In: StatPearls [Internet]*.

[28] Chappity P., Kumar R., Sahoo A. K. (2015). Heerfordt’s syndrome presenting with recurrent facial nerve palsy: case report and 10-year literature review. *Sultan Qaboos University Medical Journal*.

[29] Wessendorf T. E., Bonella F., Costabel U. (2015). Diagnosis of sarcoidosis. *Clinical Reviews in Allergy & Immunology*.

[30] De Smet D., Martens G. A., Berghe B. V. (2010). Use of likelihood ratios improves interpretation of laboratory testing for pulmonary sarcoidosis. *American Journal of Clinical Pathology*.

[31] Studdy P. R., Lapworth R., Bird R. (1983). Angiotensin-converting enzyme and its clinical significance--a review. *Journal of Clinical Pathology*.

[32] Mahévas M., Lescure F. X., Boffa J. J. (2009). Renal sarcoidosis. *Medicine*.

[33] Scadding J. G., Mitchell D. N. (2013). *Sarcoidosis*.

